# 

**Published:** 1856-01

**Authors:** 


CONTENTS—ORIGINAL COMMUNICATIONS.

Treatment of Dental Caries when complicated with an Irritable or Exposed Pulp—Continued. By James Taylor....113
Humbugiana. By One who has been "Sold."....122
Correspondence. N. B. Slayton....129

SELECTED ARTICLES.

Local Anæsthesia by Congelation in Dental Surgery. By J. Richard Quinton, Lon..133
Crystaline Gold. [A "Review of Editorial Remarks upon Sponge Gold, by Dr. J. D. White," in "The Dental Register of the West."] By "J. T." January, 1855..150
Extracts from the "London Lancet,"....160
Report of a Case of Nervous Disease. By Harry Dove, M. R. C. S., &c.....162
On the use of Amalgan for Filling Teeth. By Elisha Townsend....165
Executive Clemency—Pardon of Dr. Beale....171
Cases of Aveolar Abscess....173
On the Cure of Tooth-ache, and a Method of Treating Exposed Nerve. By Donaldson Mackenzie, Esq., Surgeon Dentist....179
Extracts from the Records of the Boston Society for Medical Observation. By S. P. Sprague, Secretary....186
Neuralgia Facial, or Tic Douloureux. By T. D. Thompson, D. D. S., Providence, Rhode Island....189
Three Cases from my Note Book. By S. H. Harvey, M. D., Washington, Ark......190

DENTAL ABSTRACTS.

Dental Deformities....19
Extracts from the "News Letter,"....194
Continuous Gum....200
Complete Dislocation of the Lower Jaw reduced by a New Method....201
"A Word with the Doctors," from the "Western Lancet,"....203
New Operation for Cleft Palate; Means of Counteracting the Effects of Chloroform, 198
Correspondence—Original....206
Miscellaneous Items....209
Editorial....213

